# Spinning Carbon Nanotube Nanothread under a Scanning Electron Microscope

**DOI:** 10.3390/ma4091519

**Published:** 2011-08-29

**Authors:** Weifeng Li, Chaminda Jayasinghe, Vesselin Shanov, Mark Schulz

**Affiliations:** Nanoworld Laboratories, University of Cincinnati, Cincinnati, OH 45221-0072, USA; E-Mails: jaya.chaminda@gmail.com (C.J.); shanovvn@ucmail.uc.edu (V.S.)

**Keywords:** carbon nanotubes, manipulation, nanothread, nanotechnology

## Abstract

Nanothread with a diameter as small as one hundred nanometers was manufactured under a scanning electron microscope. Made directly from carbon nanotubes, and inheriting their superior electrical and mechanical properties, nanothread may be the world’s smallest man-made fiber. The smallest thread that can be spun using a bench-top spinning machine is about 5 microns in diameter. Nanothread is a new material building block that can be used at the nanoscale or plied to form yarn for applications at the micro and macro scales. Preliminary electrical and mechanical properties of nanothread were measured. The resistivity of nanothread is less than 10^−5^ Ω∙m. The strength of nanothread is greater than 0.5 GPa. This strength was obtained from measurements using special glue that cures in an electron microscope. The glue weakened the thread, thus further work is needed to obtain more accurate measurements. Nanothread will have broad applications in enabling electrical components, circuits, sensors, and tiny machines. Yarn can be used for various macroscale applications including lightweight antennas, composites, and cables.

## 1. Introduction

Carbon Nanotubes (CNT) raise the expectation of replacing gold and copper in electrical applications, replacing steel and alloys in mechanical applications, and replacing diamond in thermal applications. These advances depend on solving technical challenges with handling nanotubes and scaling them up to bulk materials. At the microscale, the difficulty is manipulation. At the macroscale, the difficulty is proportionally magnifying the CNT properties. An approach to transition from nanotubes to macroscale thread is shown in this paper. If scalability of carbon nanomaterials is achieved, transitioning to a carbon industrialized society is envisioned. Carbon industrialization would replace metals, silicon electronics, and conventional fibers with nanotube based materials including yarn, fabric, braid, and sheet. Carbon engineered piping, cables, airframes, automobiles, electric motors, and electronics would replace existing incumbent designs.

In the materials world, smaller is better because smaller size materials have fewer defects and quantum characteristics. On the other side of the fence, macroscale materials are needed for engineering applications. This disparity in scale has caused a long-standing challenge in the design of man-made materials—How can nanostructure be incorporated into macroscale materials to obtain the most advantageous properties? Looking to nature for a solution, hierarchical design is used in different natural materials such as bone to transition from microscale features to macroscale size. Thus transitioning from tiny carbon nanotubes to macroscale carbon nanotube thread might be achieved in a step-wise graded manner. CNTs are grown in arrays or forests that can be mm in length. Nanotubes in the forest can be pulled and twisted to form thread. The smallest diameter thread (e.g., 5 microns minimum) spun on a bench-top machine has the greatest strength (force/tube cross-sectional area).

A grand challenge of nanotechnology research around the world is to improve the properties of nanotube thread. It has been shown [1] that the properties of thread will increase as the diameter of thread decreases. Small diameter thread has an advantage in that it can have more turns per unit length for a given helix angle as compared to larger diameter thread. More turns means more radial grip and better properties. Also stress is more uniform across smaller diameter thread. This holds for thread that is above a minimum diameter and length that allows thread to function as thread. When the diameter of thread becomes too small, the number of CNTs inside the thread becomes small, and the fluctuation effect will be severe which will lead to non-uniformity and more defects. Thus the mechanical properties will decrease. The smallest diameter practical to form nano thread is expected to be about 100 nm. For our CNTs, the diameters are about 10 nm. This means there are about 80 CNTs in the cross sectional area of a 100 nm diameter thread which should be enough to function as a thread. The challenges are that small diameter thread is difficult to spin or handle, and no one has been able to produce thread in the nanometer diameter range. To solve this problem, a hierarchical strategy is adopted to form macroscale thread. The first step is to produce small diameter thread or “Nanothread.” This was attempted by spinning carbon nanotube arrays into thread under a scanning electron microscope (SEM) using robotic manipulators. Then the properties of this thread were evaluated. Showing improvement, the process to manufacture yarn was then considered. Scaling up is possible by plying the small threads together to form a macroscale yarn. Yarn can also be plied to form larger ropes. This paper describes how the first man-made nanothreads were created.

## 2. Experiments and Results

CNTs used to make nanothreads are grown by the chemical vapor deposition (CVD) method in our lab. They are about 10 nm in diameter and 500 µm in length. The maximum current that a single CNT can carry is about 30 µA. The resistivity is as small as 6 × 10^−7^ Ω∙m at elevated temperature. The strength of a single CNT has not been tested. CNTs capable of being spun into thread are about 10 nanometers in diameter [2,3,4,5]. The CNTs have good electrical, mechanical, and thermal properties [6,7,8,9] which allows them broadly to be used in many areas. A CNT FET and CNT radio [10,11] and other nanoscale devices have already been invented. To use CNT at the macroscale, CNT arrays must be spun into thread. Through spinning, millimeter long CNT arrays can form CNT threads that can be km long. Two or more CNT threads can be plied together to form a CNT yarn which is easier to handle and use. But there are some limitations in making thread and yarn.

Kallista *et al.* [12] show a cross-section of a CNT thread under the SEM. The density of the CNTs decreases from the center to the outer surface of the thread. When the thread breaks, the center section will carry more load than the outer part. This will cause the center to break first, then the outer part. Hence, the strength of thread decreases with increasing diameter. The solution to this problem is to make small diameter threads and wrap them together to form yarn. CNT threads presently cannot be spun below about 5 µm. Below this diameter, the thread is too small to handle in a machine without it breaking and it is almost invisible to the naked eye.

In the NanoWorld Lab [13], a micromanipulator made by Kleindiek [14] is used to handle nanoscale materials. The micromanipulator has plug-in accessories that were adapted to prepare thread and characterize it. A rotational tip is used for twisting a CNT bundle into thread. Driven by a power supply outside the SEM, the piezo motor can rotate step by step for an unlimited angle. A tungsten probe was attached to the rotational tip. The probe is used to pick up a very small bundle of CNTs and twist the bundle into a thread.

**Figure 1:**
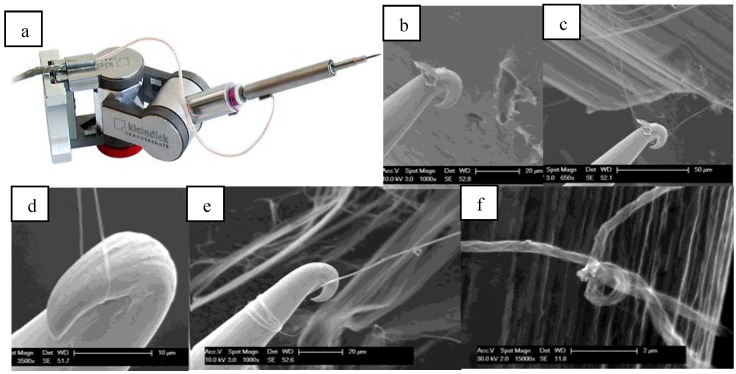
Spinning nanothread: (**a**) rotational tip used; (**b**) making a hook by pushing a probe against aluminum; (**c**) using the hook to pick up a very small bundle of Carbon Nanotubes (CNTs); (**d**) pulling and rotating the hook; (**e**) CNT thread is wound on the hook; (**f**) CNT nanothread with 300 nm diameter; The thread is so thin it looks almost transparent under the SEM.

The main force acting between the nanotubes is the van der Waals force. This force allows CNT bundles to be pulled from a nanotube forest in a continuous strand. Entanglement of nanotubes also provides coupling between nanotubes to allow spinning. Due to the difficulty of using a probe tip to grab the nanotubes, a hook was formed at the end of the probe to pull and twist nanotube bundles (Figure 1). Theoretically, with this approach a thread with diameter close to the diameter of individual nanotubes could be made. In reality, if the bundle is too small, it breaks very easy. The smallest diameter thread that can be spun is about 100 nm. The thread was made by continuously rotating and pulling the hook using manual control (Figure 1).

**Figure 2:**
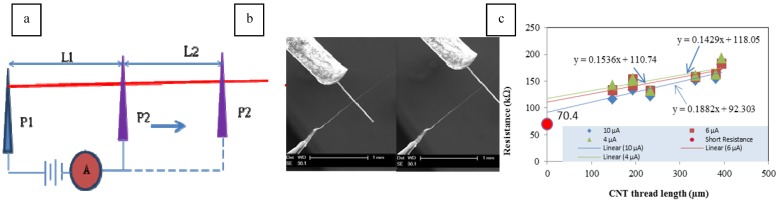
Experimental setup for measuring electrical resistance: (**a**) sketch of measurement method; (**b**) sliding the probe along the nanothread; (**c**) graph used to determine contact resistance.

Compared to a single CNT, nanothread is larger, straighter, and easier to manipulate. This offers the opportunity to measure resistivity and to remove the contact resistance. Resistivity testing was performed using the Kleindiek Low Current Measurement Kit (LCMK). Two probes are used in the measurement. During the experiment, probe one (P1) is fixed and probe two (P2) is slid along the thread (Figure 2).

Theoretically, two measurements made at different positions can be used to determine the contact resistance and the resistance of the thread material. Let the external voltage be *V*, circuit resistance (resistance from probes, wiring, and electrical devices) be *Rc*, contact resistance between probes and the CNT sample be *Rcontact*, resistivity be *ρ*, and cross-sectional area be *A*. At the two positions, currents *I1* and *I2*, and lengths *L1* and *L2* will be measured. The following equations are used to compute the two resistances:

(1) $$\frac{V1}{I_{2}}=Rc+Rcontact+\rho \frac{L1+L2}{A}$$

(2) $$\frac{V2}{I_{1}}=Rc+Rcontact+\rho \frac{L1}{A}$$

Subtracting (1)–(2) gives

(3) $$\frac{V1}{I_{2}}-\frac{V2}{I_{1}}=\rho \frac{L2}{A}$$

Only *ρ* is unknown in Equation (3). To decrease the error in the experiment, measurements were made at several different positions. Resistivity measurement was done on a 300 nm diameter thread. In this experiment, the contact area is very small (the thread is located near the ends of the tips) and the current also passes through the rotational tip. The circuit resistance was 70.4 kΩ measured directly by connecting two probes. Three different currents (4 µA, 6 µA, 10 µA) were applied to the sample to reveal possible current dependence. Contact resistances for these three tests were 48 kΩ, 40 kΩ, 22 kΩ from Figure 2(c). These contact resistances were subtracted from original data to calculate resistivity. Resistivity from this experiment is about 1.2 × 10^−5^ *Ω·m for as grown nanotubes* in bundles. Annealed CNT bundle samples were also tested. CNT annealing was done in a high temperature furnace at the Air Force Research Laboratory in Dayton, OH. Annealing was done by purging the chamber of oxygen and refilling with argon gas multiple times and final heating for one hour at 2500 °C. When large currents passed through the already annealed bundle samples, the sample becomes hot and glows in a vacuum and resistivity as small as 6 × 10^−7^ *Ω·m* was measured.

A force measurement tip (FMT) from Kleindiek was used and needs to be carefully aligned and calibrated. SEM glue was used to fix the samples onto the FMT and probes. Electron Beam Induced Deposition (EBID) is another approach to attach single or very small bundles of CNTs, but it is not practical for the relatively larger nanothreads tested here. Several steps are required to perform a test. First, using SEM glue, one end of the sample is attached to the FMT. Second, the other end is attached to a moving probe. Third, the probe is moved to break the sample. The results are recorded by Kleindiek software in a computer.

Different CNT samples were tested and the results are listed in Table 1. The terminology used for describing the samples is explained. A strand is made by pulling a thin layer of CNT from the array without twisting. The strand can be continuous. There are junctions in the strands where the nanotubes overlap. A bundle is made by pulling CNT from the array and is usually the length of the nanotubes in the array. It has no junctions and no twisting. For the strand and bundles, the diameter was measured directly from the SEM. These diameters are used to compute the cross-sectional area of the thread. This area is larger than the actual cross-sectional area of all the nanotubes because there is space between the nanotubes in the array and in the strand/bundle.

**Table 1:** Measured force and approximated strength for CNT samples based on the CS area of the tubes.

Type	Length (µm)	Diameter (µm)	Force (µN)	Strength (GPa)
Strand	2000	2	15	0.1
Twisted Strand (Thread)	300	0.4	70	0.5
Bundle	25	1	300	4.8
Twisted Bundle	275	0.4	300	2.4

## 3. Discussion

Experiments in which bundles were condensed using a solvent indicate the diameter of twisted samples is roughly ¼ of the diameter of directly measured untwisted samples. Thus the real strength of the strand and bundles should be roughly 16 times larger than based on the diameter of the loose material. Using the approximated diameters (removed empty space between CNTs), the strength of a strand is about 0.1 GPa. After twisting, the strength of the strand (now a thread) increases to 0.5 GPa. Thus twisting increases the strength by five times. The strength of bundles is about 4.8 GPa. This value is two times as large as the strength of the ‘Twisted Bundle’. These two results show an interesting conclusion: Twisting improves the strength of junctions where the nanotubes overlap, but reduces the strength of straight CNTs. Thus, don’t twist CNT if there are no junctions. Experience from microthread shows 30° is the best twist angle. In the SEM, this twisting is hard to control. But is it desired to make the twist angle close to 30°.

Comparing micron diameter threads and nano diameter threads, nanothreads have several advantages and one disadvantage. For the same twist angle, nanothreads have more turns per unit length than normal threads. This means there will be more twisting over the junctions where the nanotubes overlap if the CNTs in microthreads and nanothreads have the same length of overlap. Testing the strength of strands shows the strength of the junction is very low. In strands, the parallel van der Waals force is the main force available to make the connection. If the junction is twisted, a normal force pointing to the center of the thread will be created by the outer layer of CNTs. Hence, a friction force will also exist to prevent the CNTs from sliding apart.

The other advantage is that the variation in spatial density of nanotubes throughout the cross section is reduced for nanothread. In microthread, CNTs in center are not twisted as much as the outer tubes. The straighter load path causes the center CNTs to break first, then the outer CNTs fail. Ideally, the CNTs in nanothread should fail uniformly to achieve maximum strength. Observation under SEM shows the failure mechanism of nanothread is the CNTs sliding apart. The van der Waals force plus the static friction force is not large enough to break the CNTs. This is the disadvantage. One solution to this problem is to spin longer CNT into thread. This approach is being investigated. Another solution is to spin two nanothreads into a larger yarn to increase the normal force and friction force. Then the two yarns will be spun together and the process can be repeated. Finally, a super strong macroscale yarn might be made. In principle, long rope-like cables [15] could be made by hierarchical assembly.

**Figure 3:**
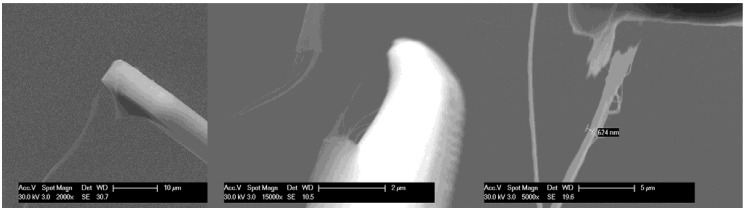
In strength tests, the breaking point is always near the SEM glue.

**Figure 4:**
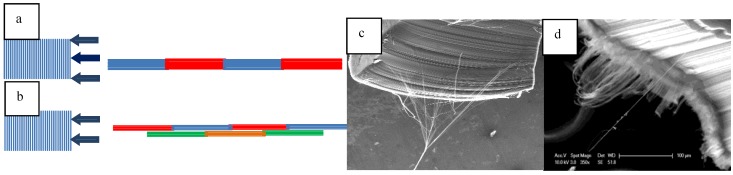
Different positions for pulling thread: (**a**) a ribbon with junctions mostly aligned which will produce weak thread; (**b**) a ribbon with junctions distributed evenly which will produce strong thread; (**c**) thread formed using the distributed method top to bottom in (**b**); (**d**) thread formed using the aligned top to bottom method in (**a**).

In the strength tests, submicron threads usually break near the SEM glue (Figure 3). This implies the thread is stronger than the glue-CNT mix. A different approach to grip the CNT is needed to provide more accurate results. Another result to be pointed out, and which became apparent only after using the SEM to observe the drawing of thread, is the importance of the drawing position on a CNT array (Figure 4). The pulling positions in Figure 4(a) will cause the nanotube to nanotube junctions to be mostly aligned. The drawing positions in Figure 4(b) will cause the junctions to be staggered and distributed. After twisting, nanothread made as shown in Figure 4(b) is much stronger than in Figure 4(a). This size effect is not much of a problem for large diameter thread that is spun from a wide section of a forest, but is critical for spinning nanothread.

The last point is, the strengths reported here are based on the cross-sectional area of the nanotubes and thus can be used for engineering design. In some papers, the strength of nanotube materials is reported based on the cross-sectional area of the walls only, which is misleading and not useful for engineering design. By improving the post treatment of nanotubes, and developing a testing method that does not use glue which reduces the strength, we believe nanothread should be much higher than measured herein.

## 4. Conclusions

In summary, nanothreads were made and characterized under an electron microscope. Engineering properties were reported based on the cross-sectional area of the thread, and the properties can be used for design. The properties of nanothread are better than the properties of microthread, but still below the properties of individual nanotubes. Nanothread is good enough now to be a new building block for electrical components. Scale up of the nanothread to form macroscale yarn may be done by twisting the nanothread together repeatedly to form higher order hierarchal structures. This opens the possibility of putting nanotube thread into applications such as composite materials, electrical conductors, and electromagnetic devices.
